# Treatment Coverage Estimation for Mass Drug Administration for Malaria with Dihydroartemisinin–Piperaquine in Southern Province, Zambia

**DOI:** 10.4269/ajtmh.19-0665

**Published:** 2020-07-02

**Authors:** Timothy P. Finn, Joshua O. Yukich, Adam Bennett, Travis R. Porter, Christopher Lungu, Busiku Hamainza, Elizabeth Chizema Kawesha, Ruben O. Conner, Kafula Silumbe, Richard W. Steketee, John M. Miller, Joseph Keating, Thomas P. Eisele

**Affiliations:** 1 Department of Tropical Medicine, Center for Applied Malaria Research and Evaluation, Tulane University School of Public Health and Tropical Medicine, New Orleans, Louisiana;; 2 Malaria Elimination Initiative, Global Health Group, University of California San Francisco, San Francisco, California;; 3 PATH Malaria Control and Elimination Partnership in Africa (MACEPA), Lusaka, Zambia;; 4 National Malaria Elimination Centre, Zambia Ministry of Health, Chainama Hospital, Lusaka, Zambia;; 5 PATH MACEPA, Seattle, Washington

## Abstract

Mass drug administration (MDA) is currently being considered as an intervention in low-transmission areas to complement existing malaria control and elimination efforts. The effectiveness of any MDA strategy is dependent on achieving high epidemiologic coverage and participant adherence rates. A community-randomized controlled trial was conducted from November 2014 to March 2016 to evaluate the impact of four rounds of MDA or focal MDA (fMDA)—where treatment was given to all eligible household members if anyone in the household had a positive malaria rapid diagnostic test—on malaria outcomes in Southern Province, Zambia (population approximately 300,000). This study examined epidemiologic coverage and program reach using capture–recapture and satellite enumeration methods to estimate the degree to which the trial reached targeted individuals. Overall, it was found that the percentage of households visited by campaign teams ranged from 62.9% (95% CI: 60.0–65.8) to a high of 77.4% (95% CI: 73.8–81.0) across four rounds of treatment. When the maximum number of visited households across all campaign rounds was used as the numerator, program reach for at least one visit would have been 86.4% (95% CI: 80.8–92.0) in MDA and 83.5% (95% CI: 78.0–89.1) in fMDA trial arms. As per the protocol, the trial provided dihydroartemisinin–piperaquine treatment to an average of 58.8% and 13.3% of the estimated population based on capture–recapture in MDA and fMDA, respectively, across the four rounds.

## INTRODUCTION

In coordination with the National Malaria Control Centre (NMCC) of Zambia, a community-randomized controlled trial was conducted in Southern Province to evaluate the impact of four rounds of mass drug administration (MDA) or focal MDA (fMDA), where treatment was given to all eligible household members if anyone in the house had a positive malaria rapid diagnostic test (RDT).^1^ Mass drug administration is conducted community wide, whereas fMDA is conducted at the household level. A key factor driving the effectiveness of MDA for interrupting malaria transmission is population coverage.^2–6^ Programs face challenges in reporting MDA coverage because of the difficulty in knowing the population denominator in areas where accurate household lists do not exist and official census data can be outdated or incongruent with program implementation boundaries. Accurately determining the proportion of individuals who were reached and who took the treatment course is critical for the evaluation of MDA; relying on programmatic records of drug distributors alone for who was enumerated and took treatment may provide an incomplete picture of how many target houses were not visited.^7^

Many MDA programs use after treatment coverage surveys (TCS) to validate reported MDA coverage.^8–10^ These surveys primarily use a probability two-stage cluster sampling method to estimate the treatment coverage by administering a timely and concise questionnaire to households.^11^ To estimate MDA coverage, surveys collect information to estimate both the numerator—the people reporting being treated or visited by the MDA campaign—and the denominator—the total people who should have been treated or visited.

The terminology for reporting MDA coverage is heterogeneous and markedly inconsistent in the literature and limits comparison across studies.^12,13^ Given the limited reporting of malaria MDA experiences at scale in the last 20 years, the evidence base for evaluating MDA coverage and measurement methodologies is challenging.^14,15^ To date, there have been no published studies assessing methods for estimating coverage for malaria MDA as has been performed for neglected tropical diseases.^10,16–18^

Capture–recapture techniques provide an alternative method to TCS for measuring MDA coverage. To our knowledge, capture–recapture, methods that estimate a denominator by comparing the overlap of independently collected population lists for the same population,^19^ has not previously been used for estimating the denominator for an MDA program. Primarily used in ecology to estimate the size of animal populations where censuses are difficult to conduct, capture–recapture has been used to estimate human population size as far back as 1802.^20^ In public health, capture–recapture methods have been applied to assess the completeness of registers or lists used in differing data tracking systems and to estimate the number of disease events such as HIV, tuberculosis, dengue, influenza, and malaria diagnoses^20,21^; mortality^22–24^; population sizes in hard-to-reach groups^25–28^; and completeness of disease surveillance systems.^22–24,28^

Recently, satellite images have also been used to estimate population size,^25,28^ enumerate sample frames,^29–34^ target interventions such as indoor residual spraying,^35,36^ and monitor polio vaccination activities.^37^ In most MDA contexts, however, household locations are not recorded with GPS-enabled devices; however, where they do exist, the completeness of household visitation by campaign workers can be compared against an enumeration of structures in the target area from satellite images.

This article provides the results of an effort to calculate epidemiologic coverage and program reach (household coverage) of four MDA and fMDA rounds using capture–recapture methods. The capture–recapture coverage estimates are also compared with the more commonly used TCS method for estimating MDA coverage. Satellite enumeration of households in the MDA trial area was used to assess populations that were missed by both the capture–recapture and TCS methods.

## METHODS

### Data sources.

A full description of the trial has been published elsewhere.^1,7^ In summary, the trial assessed the impact of four rounds of community-wide MDA, where all household members were given dihydroartemisinin–piperaquine (DHAp) regardless of RDT results, versus fMDA, where DHAp was given to all eligible household members if anyone in the house had a positive RDT, on malaria parasite infection prevalence from November 2014 to March 2016. Data from each MDA and fMDA round were used to determine the number of households visited, total number of individuals, and testing and treatment status. Furthermore, two household surveys were conducted from April through May in 2015 and 2016 to establish the follow-up and final parasite infection prevalence in the study arms.^7^ Each survey was implemented approximately 2–3 months after the second and fourth MDA rounds, respectively, and included a TCS module with questions assessing MDA household visitation, testing, treatment, and reasons for not being visited, tested, or treated in the two preceding mass treatment rounds. All study data were collected on Android mobile phones by pairs of community health workers (CHWs) assigned to health catchment areas. A full description of treatment adherence has been published elsewhere.^38^

An independent satellite enumeration exercise using Bing Map Imagery (Microsoft, Redmond, WA) was conducted in March 2015 to identify areas completely missed by the baseline household census, household surveys, and MDA rounds. The enumeration exercise consisted of manually pinpointing and classifying all structures based on size and assumed use. A total of 121,309 structures were enumerated in the 40 intervention catchments, of which 51,599 corresponded to GPS points provided by the MDA campaign for rounds 1 and 2. The enumeration team consolidated this file to 50,364 potential household structures in the 40 catchments, where a central point in a cluster of structures was considered a household. This methodology has been described previously.^35,36^

### Data analysis: program data.

Table 1 summarizes the coverage definitions and source data. WHO coverage definitions were adapted to serve as a framework to organize the analysis of coverage and to further standardize terminology.^39^ Program coverage estimates were calculated by aggregating for each round and trial arm the number of individuals provided treatment divided by the total individuals listed by the field teams during the mass treatment campaign rounds.

**Table 1:** Summary of coverage calculation methods

Def #	Coverage type	Numerator	Denominator	Numerator data source	Denominator data source	Data available
1	Program coverage	Number treated	Total population included in the MDA campaigns	MDA campaign data	MDA campaign data	Rounds 1–4
2	Epidemiologic coverage	Number treated	Imputed population not reached + total population included in MDA campaign data	MDA campaign data	Capture–recapture estimates + MDA campaign data	Rounds 1–4
3	Survey coverage	Number treated surveyed	Total number of eligible surveyed	HH parasite surveys	HH parasite surveys	Follow-up (R2) and final (R4)
4	Program reach (capture–recapture)	Number of HH visited by a CHW for MDA	Number of HH estimated by capture-recapture	MDA campaign data	Capture–recapture estimates	Rounds 1–4
5	Program reach (survey)	Number of HH visited by a CHW for MDA	Total number of HH surveyed	HH parasite surveys	HH parasite surveys	Follow-up (R2) and final (R4)
6	Program reach (satellite enumeration)	Number of HH visited by a CHW for MDA	Satellite enumeration health facility catchment area HH	MDA campaign data	Satellite enumeration	Rounds 1–4

CHW = community health worker; HH = household; MDA = mass drug administration.

### Data analysis: capture–recapture data.

**Figure 1.**
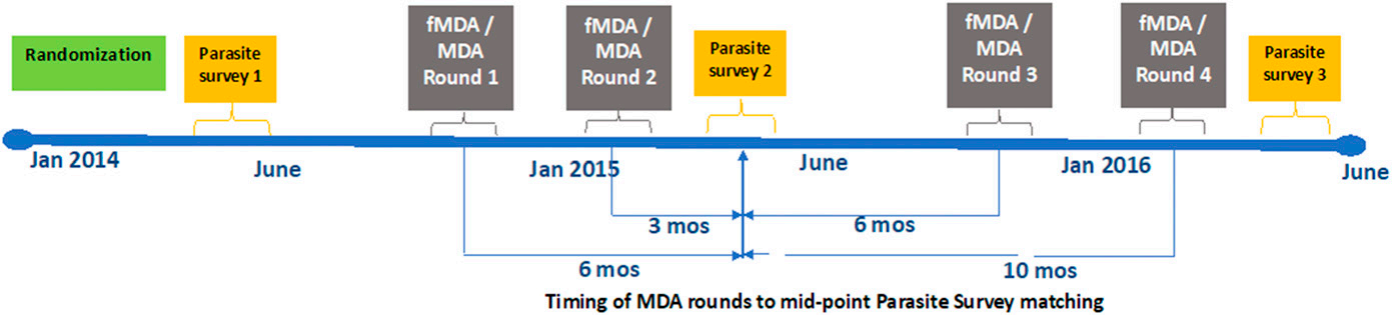
Study timeline and matching of rounds to parasite survey. This figure appears in color at www.ajtmh.org.

Capture–recapture methods were used to estimate the total number of households in each catchment that should have been visited by campaign field teams. Individuals from two independent lists—the campaign round datasets and the 2015 midpoint parasite survey—were matched using record linkage theory on the basis of name, age, gender, catchment, and household GPS coordinates.^40^ Figure 1 illustrates the timing of matched study activities. A household was considered matched from each campaign round to the survey if at least one enumerated household resident was matched. Matches were restricted to the same catchment area. The number of households per catchment was calculated using the *Schnabel* estimation methodology.^41^ There are three primary assumptions that must be met for this method: 1) the population is closed without gains or losses between mark–recapture, 2) sampling is random, and 3) all individuals have an equal chance of capture.^20^

Program reach was calculated by dividing the total reported number of households visited by the trial arm by the total number of households that should have been visited during campaign rounds based on capture–recapture. The total population not reached was imputed by multiplying the average household size for the trial area by the difference in the total number of households calculated by capture–recapture and the actual number of households visited. The denominator for epidemiologic coverage comprises the total enumerated population by round and arm and the imputed population not reached. Record matching and Schnabel analyses were performed in R version 3.3.1 (R Foundation for Statistical Computing, Vienna, Austria) and manual verification in Microsoft Excel version 15.30.^42^ For Schnabel estimation, the Simple Fisheries Stock Assessment Methods package was used to corroborate manual estimation.^43^

### Data analysis: satellite enumeration data.

Each MDA round dataset was matched to the spatially enumerated household structure file based on geo-location, excluding houses with missing GPS points. All households from each campaign round were plotted, and a 40-m buffer was drawn around the household point. If plotted satellite structures fell within a buffered household, then the satellite structure was considered matched; after each round, the balance of structures not visited was matched to the next round until all rounds were plotted and matched. The structures not matched after comparison to round four of the trial were considered not visited during any campaign round. Program reach based on satellite enumeration was calculated by dividing the total number of structures visited by campaign teams by the total structures enumerated.

### Data analysis: household survey data.

Survey coverage is the percentage of survey respondents who reported being treated in rounds two and four divided by all residents recorded in the surveyed households. The percentage of surveyed households reporting being visited for rounds 2 and 4 comprises the program reach for each round by arm. Univariate and multivariable logistic regression analyses were performed to measure whether there were differences in demographics and household characteristics among survey respondents who received an MDA visit and those who did not. Calculations were performed using Stata 13.1 (StataCorp, College Station, TX) and adjusted for the cluster survey design. Coverages were aggregated by fMDA, MDA, and both arms.

## RESULTS

### Program data.

The number of individuals and households enumerated during each campaign round decreased in rounds two and four, which occurred during the rainy season, compared with rounds 1 and 3, which occurred during the dry season (Table 2). The average number of households visited for MDA and fMDA during rounds 1 and 3 was 17,883 and 17,854, respectively, and decreased to 14,921 and 15,330 during rounds 2 and 4, respectively. Program coverage ranged from 79.9% to 87.0% for the MDA trial arm and 10.2% to 26.6% for the fMDA trial arm across the four rounds. The low coverage and decrease in fMDA accurately reflects application of the trial protocol, where only people in households where someone was RDT positive received treatment, amid declining parasite prevalence over the trial period.^7^ Adherence information was collected for 181,534 of 336,821 DHAp (53.9%) treatments administered during four rounds of MDA/fMDA, of which 153,197 (84.4%) reported completing the full course of DHAp.^38^ Approximately 340 mobile phones (three to 20 per catchment) were used during each MDA round for data collection.

**Table 2:** Treatment coverage for MDA campaign rounds–program, epidemiologic and survey

Category	Round 1		Round 2		Round 3		Round 4	
	fMDA	MDA	fMDA	MDA	fMDA	MDA	fMDA	MDA
MDA campaign data								
Total households visited	17,704	18,237	14,610	14,584	18,004	17,528	16,050	15,257
Community health worker enumerated population (a)	95,214	90,347	79,518	70,305	88,605	90,077	81,208	79,653
Total courses of dihydroartemisinin–piperaquine administered (b)	25,372	78,591	17,092	56,620	14,599	72,006	8,256	64,285
Program coverage (%)	26.6	87.0	21.5	80.5	16.5	79.9	10.2	80.7
Capture–recapture–based adjustments								
Number of households not visited	5,797	4,750	8,891	8,403	5,497	5,459	7,451	7,730
Imputed population not listed (c)	29,565	24,225	45,344	42,855	28,035	27,841	38,000	39,423
Epidemiologic coverage* (%) (d)	20.3	68.6	13.7	50.0	12.5	61.1	6.9	54.0
Survey data								
Number surveyed	–	–	1,644	2,472	–	–	1,137	2,050
Survey coverage (95% CI)	–	–	31.9% (22.0–43.8)	83.2% (77.2–87.9)	–	–	11.4% (4.2–27.6)	88.0% (85.6–90.0)

fMDA = focal MDA; MDA = mass drug administration.

* *d* = *b*/*a* + *c*.

### Capture–recapture.

The percentages of individuals from the midpoint household survey (*n* = 8,142 in 1,649 households) that were matched to individuals enumerated in campaign rounds 1 through 4 were 43.5%, 49.6%, 37.6%, and 42.1%, respectively. The resulting percentages of households from the survey where at least one matched individual from each campaign round was found were 70.4%, 78.6%, 66.3%, and 70.8%, respectively, by round. For a sensitivity analysis, considering the 9- to 10-month lag between the midpoint household survey and round 4, the final household survey that occurred 2–3 months after round 4 was matched with the same methods. Results were comparable with 47.1% of individuals and 76.8% of households matched.

The total number of households calculated in the 40 catchment areas was 46,407 (95% CI: 44,345–48,671) (Table 3). The maximum number of reported households visited for all catchments in any round by CHWs was 39,482. The total number of households from capture–recapture was 17.5% greater than the maximum number of households visited in any round and 5.2% less than the Ministry of Health administrative population estimates (*n* = 48,938).

**Table 3:** Estimated household program reach by campaign round and trial arm from capture–recapture

Trial arm	Estimated households	Round 1, % (95% CI)	Round 2, % (95% CI)	Round 3, % (95% CI)	Round 4, % (95% CI)	Zambia census	Trial maximum visited
Focal MDA	23,501	75.3 (70.3–80.3)	62.2 (58.0–66.3)	76.6 (71.5–81.7)	68.3 (63.8–72.8)	25,774	19,625
Mass drug administration	22,987	79.3 (74.2–84.5)	63.4 (59.3–67.6)	76.3 (71.3–81.2)	66.4 (62.0–70.7)	23,164	19,857
Total	46,407*	77.4 (73.8–81.0)	62.9 (60.0–65.8)	76.6 (73.0–80.1)	67.5 (64.3–70.6)	48,938	39,482

* Estimated households differ at differing levels of aggregation.

**Figure 2.**
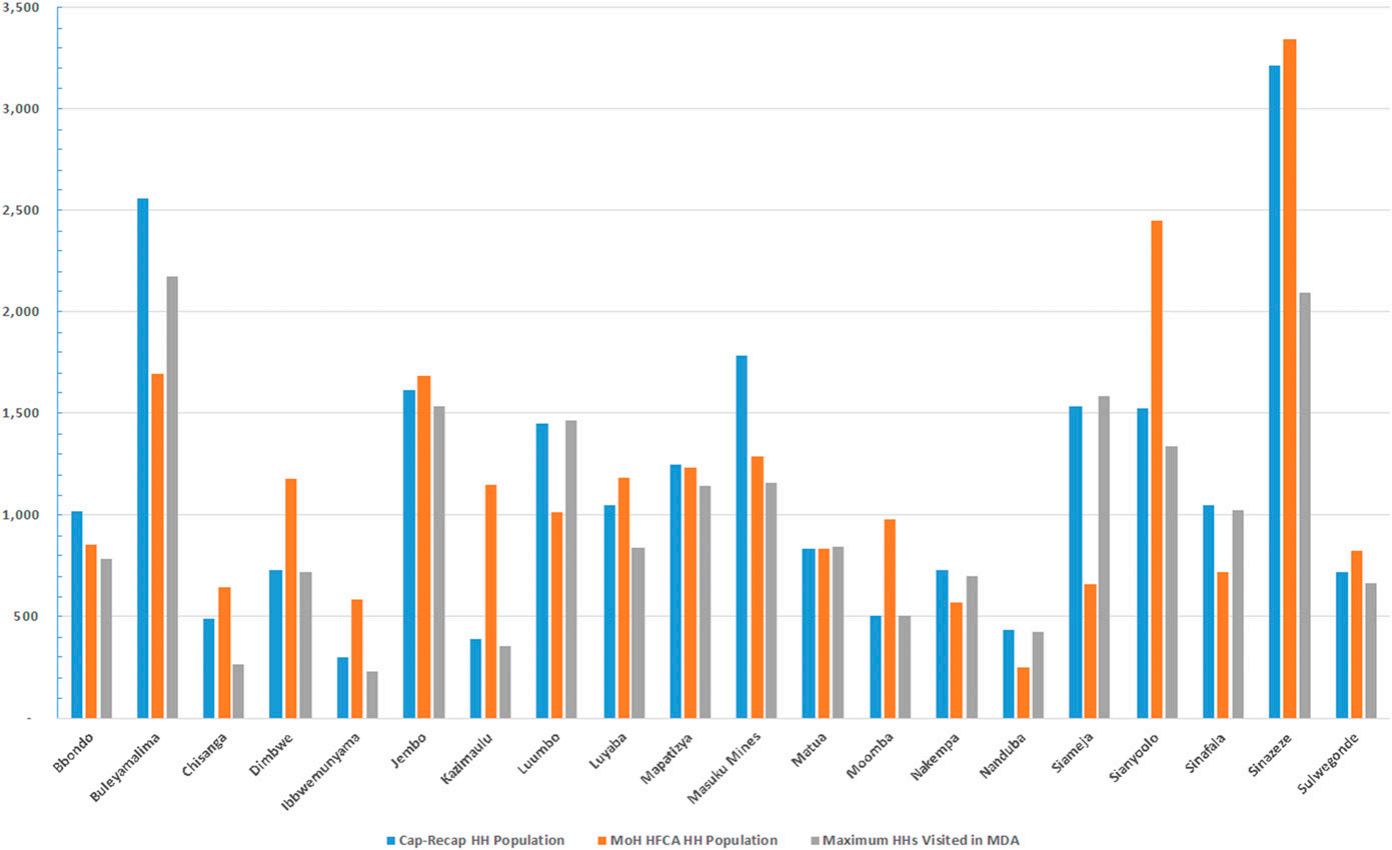
Comparison of capture–recapture, Ministry of Health administrative and maximum campaign round households per catchment for the mass drug administration trial arm. This figure appears in color at www.ajtmh.org.

**Figure 3.**
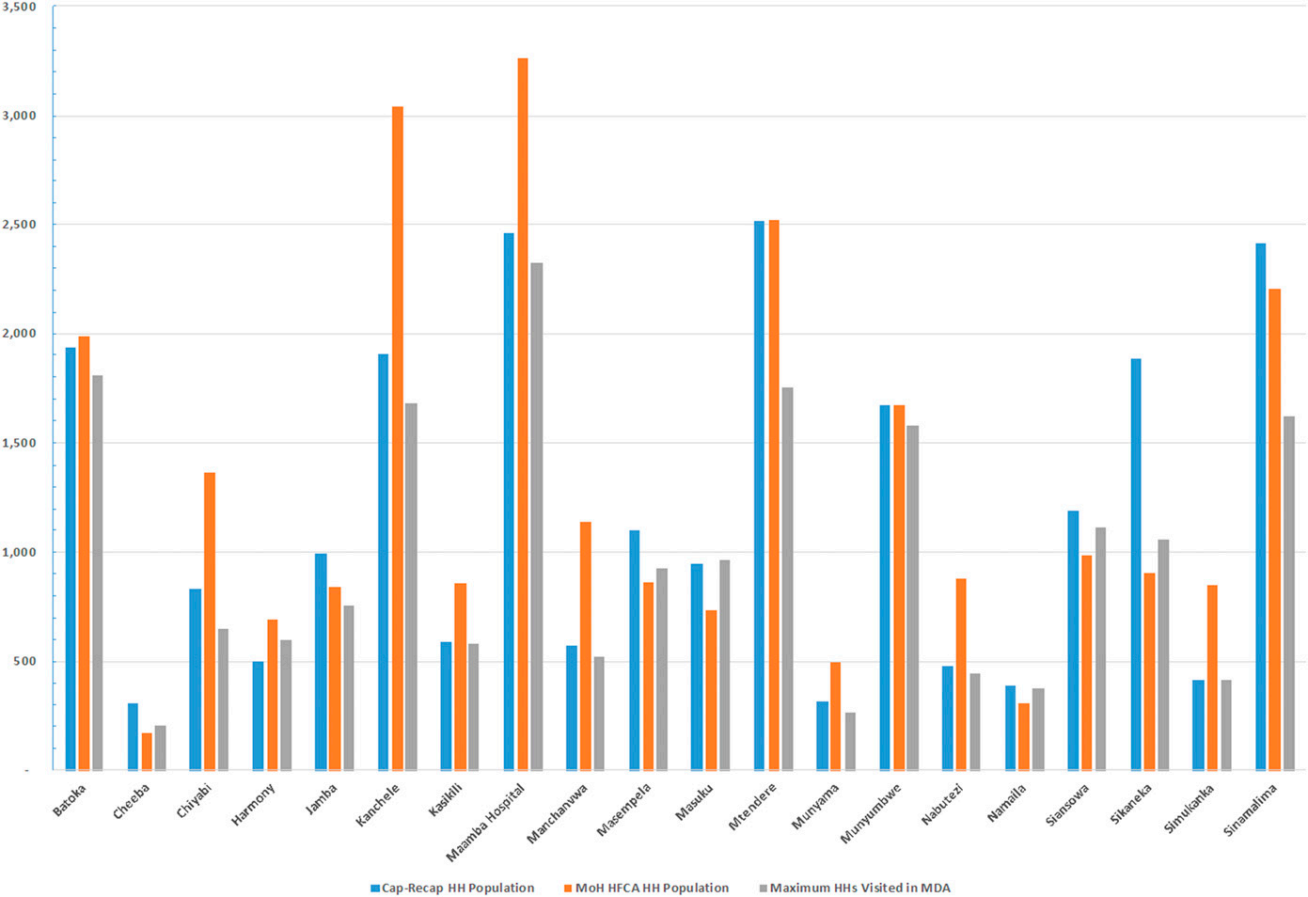
Comparison of capture–recapture, Ministry of Health administrative and maximum campaign round households per catchment for the focal MDA trial arm. This figure appears in color at www.ajtmh.org.

Table 3 summarizes the program reach (i.e., household coverage) by trial arm and round using the capture–recapture method. The percentage of households reached in MDA ranged from a low of 63.4% (95% CI: 59.3–67.6) in round 2 to a high of 79.3% (95% CI: 74.2–84.5) in round 1. For fMDA, results were similar with a low in round 2 of 62.2% (95% CI: 58.0–66.3) to a high of 76.6% (95% CI: 71.5–81.7) in round 3. Overall, if the maximum number of visited households across all campaign rounds was used as the numerator, then the program reach for at least one visit would have been 86.4% (95% CI: 80.8–92.0) in MDA and 83.5% (95% CI: 78.0–89.1) in fMDA trial arms. Figures 2 and 3 illustrate variation in the household estimates from capture–recapture, campaign workers, and national estimates at the catchment level. Review of the study area and matching results demonstrated pockets of catchments that were never visited by campaign teams; principally in Namaila, Mapatizya, Luyaba, Mtendere, and Nanduba catchments.

Based on capture–recaptures results, the reported program coverage was adjusted by the imputed number of individuals in the estimated number of households not visited (Table 2) to calculate epidemiologic coverage. For MDA, the epidemiologic coverage ranged from 50.0% to 68.6%. Declines were similar in rounds 2 and 4 (done during rainy seasons) as compared with rounds one and three (dry seasons) based on difficult logistics during the rainy seasons, as discussed earlier.

### Satellite enumeration.

A total of 50,634 probable household structures were enumerated, of which the percentage of structures visited in any round of MDA and fMDA by campaign teams was 68.4% (95% CI: 62.5–74.3) and 64.8% (95% CI: 58.1–71.5), respectively. This compares to an average of 71.1% from capture–recapture that were visited across all four rounds. The remaining structures not matched (16,303) were not verified as being occupied or valid dwellings. GPS points were missing for 15.6% of households in rounds 1 and 2 and 6.9% in rounds 3 and 4.

### Survey data.

In the MDA trial arm, the percentage of survey households being visited during rounds 2 and 4 was 72.2% (*n* = 759, 95% CI: 62.4–80.2) and 83.4% (*n* = 869, 95% CI: 74.6–89.6), respectively. In the fMDA arm, the percentage of survey households being visited during rounds 2 and 4 was 53.9% (*n* = 729, 95% CI: 44.6–63.0) and 66.2% (*n* = 837, 95% CI: 52.0–78.0), respectively. Each survey estimate of program reach was greater than the program reach estimates based on capture–recapture and satellite enumeration besides round 2 of fMDA (Table 4). Table 1 presents comparison of program and survey coverages.

**Table 4:** Comparison of program reach estimates for MDA and fMDA rounds 2 and 4 from survey, capture–recapture, and satellite enumeration

Category	MDA round 2	MDA round 4	fMDA round 2	fMDA round 4
	Prop (95% CI)	Prop (95% CI)	Prop (95% CI)	Prop (95% CI)
Household surveys, *n*	755	896	725	851
Any visit by a CHW for MDA	0.89 (0.84–0.93)	0.90 (0.84–0.95)	0.63 (0.54–0.72)	0.74 (0.61–0.88)
Of HH visited, reporting two HH visits	0.76 (0.66–0.84)	0.45 (0.27–0.63)	0.75 (0.67–0.83)	0.55 (0.41–0.69)
Program reach*	0.72 (0.62–0.80)	0.83 (0.76–0.91)	0.54 (0.45–0.63)	0.66 (0.53–0.79)
Capture–recapture, *n*	23,197	23,197	23,413	23,413
Program reach	0.63 (0.45–0.81)	0.66 (0.47–0.85)	0.62 (0.44–0.81)	0.69 (0.48–0.89)
Satellite enumeration, *n*	24,574	24,574	24,960	24,960
Program reach by a CHW at any time during trial	0.65 (0.58–0.71)		0.68 (0.62–0.74)	

CHW = community health worker; fMDA = focal MDA; MDA = mass drug administration.

* Coverage data were not collected for rounds 1 + 3.

## DISCUSSION

This study used multiple methods to calculate and triangulate the coverage of MDA and fMDA interventions across four treatment rounds in a community-randomized controlled trial. The trial provided 336,821 doses of DHAp to 674,927 individuals, as per protocol, over four rounds, representing a massive effort to reduce the parasite reservoir. Results from the household surveys examining treatment, visitation, testing, and RDT positivity after two of the four rounds suggest campaign staff program data were accurate and reliable, with differences being minor. However, relying exclusively on the program data for calculating coverage of DHAp and program reach would unknowingly lead to biased results. These methods do not inform who was potentially not visited and not offered treatment. The program data from this trial unequivocally established that many individuals were not treated during rounds 2 and 4, which were during the rainy season. Trial implementers noted the decrease in the number of households visited after round 2 and commissioned satellite enumeration to better understand the number of potential households and the totality of unvisited areas. Capture–recapture was used after the trial to estimate the total number of households residing in the trial area that were potentially not visited and provided DHAp and used to estimate epidemiologic coverage.

Capture–recapture and satellite enumeration estimates of program reach were comparable to each other but lower than those relying solely on campaign data from CHWs. Based on a fixed household denominator for each trial arm from capture–recapture, the trial program reach was less than household survey estimates for rounds 2 and 4 for MDA and round 4 for fMDA, whereas it was greater for round 2 for fMDA, within the bounds of the CI for capture–recapture. When examined together, capture–recapture and satellite enumeration methodologies provided two estimates of the total number of households within which the 2010 official adjusted Zambian census estimate was the midpoint. When comparing each with the maximum number of households visited by campaign staff during any round in each catchment, these findings affirm that many households were either not visited or unavailable for testing and treatment during the campaign rounds. If plotted and overlapped with previous catchment boundaries from other work in the region, select areas of catchments that were not visited may have been misunderstood as being outside the trial implementation boundaries and erroneously excluded by campaign staff.^44^ Attempts to confirm structures as unoccupied in later rounds were inconsistent, and structures could not be reliably removed from the datasets before analysis. Although similar work in Zambia and Malawi has demonstrated the method’s accuracy to correctly predict sleeping structures, without conclusive evidence that the structures enumerated herein had occupants at the time of the round visits, it is difficult to unequivocally affirm the precision of the capture–recapture population.^31,34,45^ Therefore, these coverage estimates are conservative, and actual program reach and epidemiologic coverage is likely greater.

These results illustrate the challenges community-based MDA programs face when calculating program reach and determining how to report epidemiologic coverage. This raises questions as to what the appropriate denominator should be to determine program reach and epidemiological coverage over a lengthy implementation period. Recent studies examining the variation in subnational populations highlight the importance of examining this issue further; relying on static denominators may overestimate the incidence of malaria by 30% because of substantial population movement.^46^ Other studies in this geographic area have also noted variation in intervention coverage by rounds and that subareas are visited consistently or not at all from round to round.^5^ Additional work has highlighted the inaccuracies of denominator data and the need for refined satellite enumeration and ground confirmation that accounts for migration and seasonal variation.^47,48^

In areas with dynamic population denominators, the challenge of providing complete coverage to all eligible individuals requires innovative strategies and improved MDA practices. Exploration of seasonal shifts of residents due to farming and nontraditional settings such as fish camps and boarding schools is needed to better inform program planning and to ensure that groups of individuals are not migrating to areas not covered by mass treatment and consequently at risk of serving as parasite reservoirs to areas previously treated. Furthermore, understanding how many potentially occupied structures are in a geographic target area and ensuring that they are visited each time to confirm that no one is residing there are paramount for consistent and reliable reporting. Adopting this as the best practice would address concerns over different denominators by rounds (which occurred during this trial) and better inform the appropriate MDA program reach.

Mass drug administration programs should consider additional studies of the feasibility of capture–recapture methods to ascertain its reliability and usefulness at estimating population. Although it is feasible to link structures over time through record linkage or spatial matching, there are limitations from data quality and degree of missing data, and these methods would not be available to the vast majority of MDA programs that do not use GPS-enabled devices and are administered by local health facility teams with paper registers. To this extent, satellite enumeration is a worthwhile expenditure if performed in advance of MDA activities and accompanied by micro-planning for campaign workers and ground-verification exercises.^37^ Recent work in Northern Nigeria for monitoring geographic coverage of polio campaign activities demonstrates the feasibility of alternate monitoring strategies and the benefits of the extensive use of mapped settlements with population estimates and GPS tracking of workers.^37,49^ Furthermore, these methods have been extended to new contexts, given the lack of consensus mapping of catchment target areas.^50^

### Limitations.

This work primarily focused on examining the extent to which households in the study area may not have been visited by campaign staff. These results must consider several limitations. First, with respect to capture–recapture methods used herein, in this study, the household survey population that served as the “mark” was randomly selected from an enumeration list that predated the first campaign round. Data collectors for the campaign treatment rounds were not the same individuals as the survey workers and campaign teams worked independently in their catchments and were to provide blanket coverage to all resident households. Thus, we considered assumptions of randomness and equal selection met. With respect to a closed population, as assumed in other population-level uses of closed capture–recapture models, given the short time frame in which the data capture events occurred, we considered this met.^21,23^ Although the data demonstrate that the population did change from round to round, the record linkage process was designed to find individuals from the survey houses, which were confirmed as present during April and May midway through the trial, and flag the household as having been visited.

There are several limitations to relying on the TCS as a reference comparison. First, survey data were derived from responses by the head of the household, or adult representative, on behalf of all individuals in the house approximately 2–3 months after the drug distribution depending on the catchment. Proxy status was not recorded for testing and treatment data; thus, the accuracy of these responses may be affected by recall and response biases, particularly in fMDA where RDT positivity was low and estimates of program reach were consistently lower than in MDA. Although the recent literature suggests recall may be accurate up to 6–12 months after MDA,^51,52^ the TCS module was bundled within a large, complex survey that took 1–2 hours to administer, which is not ideal for assessing coverage alone. Notwithstanding these limitations, this methodology was the most robust post-distribution coverage survey design in this trial and affirmed DHAp provision, RDT testing, and RDT positivity for the trial arms.

During large-scale trials or programs relying on electronic data capture, loss of information from hardware failure is possible and data entry quality may vary markedly by enumerator. In this trial, approximately 340 phones were used during each MDA round for household visits. Familiarity and improvements to the software led to decreased information loss for treatment data and geo-location information for structures visited in later rounds but ranged from 6.9% to 15.4% by round. Thus, the actual treatment coverage may be greater than what the programmatic records indicate in certain catchments. For a sensitivity analysis of structural matching, a 50-meter buffer increased matching from 67.6% to 71.0% and for a 75-meter buffer to 76.1%. These issues may also have affected the success of satellite enumeration matching and capture–recapture estimation biasing results downward.

## CONCLUSION

As MDA for malaria moves from trial conditions to routine administration under certain programmatic and transmission circumstances, malaria MDA programs must consider methods for reporting and validating treatment coverage and program reach similar to other intervention programs. Reaching individuals in a campaign is only one part of a process that must also include adherence to the treatment course for ensuring that the effective drug coverage is as high as possible. Known issues in relying on program coverage across numerous MDA programs from decades of MDA experience must be taken into account before launching large-scale malaria MDA activities. The NMCC manages a robust, multifaceted malaria control program, but this study highlights challenges encountered even under clinical trial conditions. With reticence toward using MDA as an elimination strategy well founded in the documented experience of its failure and its contribution to drug resistance, malaria MDA programs should consider the utility of satellite enumeration for planning MDA campaigns, particularly in areas where it has not occurred before, to aid in the segmentation of administrative areas for implementation and assessing the number of potential households.
